# Why Are Health Care Interventions Delivered Over the Internet? A Systematic Review of the Published Literature

**DOI:** 10.2196/jmir.8.2.e10

**Published:** 2006-06-23

**Authors:** Frances Griffiths, Antje Lindenmeyer, John Powell, Pam Lowe, Margaret Thorogood

**Affiliations:** ^2^Aston UniversityAston TriangleBirminghamUK; ^1^Health Sciences Research InstituteWarwick Medical SchoolUniversity of WarwickCoventryUK

**Keywords:** Internet, intervention studies, literature review

## Abstract

**Background:**

As Internet use grows, health interventions are increasingly being delivered online. Pioneering researchers are using the networking potential of the Internet, and several of them have evaluated these interventions.

**Objective:**

The objective was to review the reasons why health interventions have been delivered on the Internet and to reflect on the work of the pioneers in this field in order to inform future research.

**Methods:**

We conducted a qualitative systematic review of peer-reviewed evaluations of health interventions delivered to a known client/patient group using networked features of the Internet. Papers were reviewed for the reasons given for using the Internet, and these reasons were categorized.

**Results:**

We included studies evaluating 28 interventions plus 9 interventions that were evaluated in pilot studies. The interventions were aimed at a range of health conditions. Reasons for Internet delivery included low cost and resource implications due to the nature of the technology; reducing cost and increasing convenience for users; reduction of health service costs; overcoming isolation of users; the need for timely information; stigma reduction; and increased user and supplier control of the intervention. A small number of studies gave the existence of Internet interventions as the only reason for undertaking an evaluation of this mode of delivery.

**Conclusions:**

One must remain alert for the unintended effects of Internet delivery of health interventions due to the potential for reinforcing the problems that the intervention was designed to help. Internet delivery overcomes isolation of time, mobility, and geography, but it may not be a substitute for face-to-face contact. Future evaluations need to incorporate the evaluation of cost, not only to the health service but also to users and their social networks. When researchers report the outcomes of Internet-delivered health care interventions, it is important that they clearly state why they chose to use the Internet, preferably backing up their decision with theoretical models and exploratory work. Evaluation of the effectiveness of a health care intervention delivered by the Internet needs to include comparison with more traditional modes of delivery to answer the following question: What are the added benefits or disadvantages of Internet use that are particular to this mode of delivery?

## Introduction

The Internet is still a relatively new medium for seeking and delivering health care, although this use is increasing rapidly [1,2] and includes health information seeking [3], Internet-based peer support groups [4], online health consultations [5], and delivery of health interventions [6]. Some pioneer researchers have published studies that evaluate health interventions delivered directly to the users via the Internet for their ability to improve the health status of their users. In this paper we review their work, focusing on the reasons why these authors chose to use the Internet for delivery of a health care intervention. Our aim was to consolidate the findings from these early research papers to inform the development of future research. We include only health interventions in which the networking provided by the Internet is a component of the intervention. This is to distinguish them from other media such as print material, CD-ROM, and video. We reflect on the drivers to using the Internet for the delivery of health care. This paper does not review the outcomes of the interventions.

## Methods

### Identification of Studies

The initial identification of studies used five sources: three existing systematic reviews of eHealth interventions [7-9], a hand search of JMIR (vol 1(1) to vol 8(1)), and our own previous qualitative review of the literature concerning the Internet and consumer health information [10]. This latter review involved collation and identification of relevant literature through systematic searches of electronic bibliographic databases covering health and social sciences literature (1990 to December 2003, including Medline, HMIC, CINAHL, Sociological Abstracts, Sociofile, and Web of Science). We used search terms such as “Internet,” “electronic mail,” “computer communication networks,” and “health information,” “communication,” or “health informatics.” Two investigators reviewed the list to identify potentially relevant articles. We worked in pairs, reviewing the search results to identify relevant intervention studies. We did not set out to identify every published eHealth intervention paper, but aimed to search the majority of the available literature in a systematic way for a meaningful overview of the field.

### Inclusion and Exclusion Criteria

Inclusion and exclusion criteria were applied to the studies identified from the three sources described above. We included only peer-reviewed full research papers. We defined intervention studies as the use of information and communication technology to deliver a specific health intervention to a client or patient group. The study had to include a health-related outcome as part of evaluating the intervention, and the intervention had to include use of the networking provided by the Internet. Networked features included the use of email to contact a therapist, the use of chat rooms or bulletin boards by client/patient groups, or the transfer of personal health data via the Web between a health care site and personal network access (eg, between a clinic and patient’s home). Studies with no networked features, such as computer-based decision support systems delivered from a CD or interventions where there was no use of the Internet beyond delivery (ie, they could have been delivered by a CD), were excluded. A further key characteristic of the Internet is its accessibility via a networked computer anywhere and anytime. Hence, we excluded studies in which access to the intervention was provided only in the clinical setting as use of the intervention is restricted in place and time. It is also possible that effectiveness may be influenced by the clinical setting.

Our review focused on the use of the Internet for delivery of the interventions and therefore did not include non-Internet based telemedicine studies. The focus was on specific interventions for specific health problems, so we excluded interventions involving the provision of general Internet access such as home computers, Internet kiosks, or training in use of the Internet even if the outcomes included health related measures. We only included interventions in which the individuals using them were known to the health care professional or organization delivering the intervention to be sure that the participants were using their real identity and responding in a genuine way to the intervention. This cannot be ensured for a study that recruits participants solely via the Web, with no direct contact between investigators and participants. We excluded studies that solely involved the placing of health information on the Web for public access, even when there was opportunity for interaction or feedback.

### Analysis

When there were several papers concerning the same intervention (eg, a pilot study followed by a full evaluation), we grouped these papers together and treated them as one study. For each study, all the reasons given for delivering the health care intervention on the Internet were listed. These were the reasons the authors of the papers gave for choosing the Internet as the mode of delivery, rather than post hoc reasoning given in the discussion of the study results. We then categorized the reasons; one study could be categorized in a number of different groups. Again, we worked in pairs, comparing results and resolving any discrepancies through further examination of the papers and discussion among team members.

## Results

### Types of Interventions

We found full evaluations of 28 interventions and a further 9 interventions for which only pilot work had been published (Multimedia Appendix). All the papers were from Europe, North America, or Australia. The interventions were aimed at a wide range of conditions, including cancer (3 studies), HIV/AIDS (3 studies), diabetes (3 studies), mental health (1 study), eating disorders (2 studies), and back pain (1 study). Some targeted health promotion issues such as smoking cessation (1 study), physical activity (1 study), and obesity (3 studies). Other interventions aimed to support caregivers, for example caregivers of people with Alzheimer’s disease (3 studies), stroke patient caregivers (1 study), new or young mothers (2 studies), and parents of children in intensive care (1 study). One intervention aimed at supporting rural women with chronic illness. One study reported the delivery of cognitive behaviour therapy (CBT) for a number of disorders, including headache, tinnitus, and panic disorders; two other studies reported CBT delivery for depression and one for post-traumatic stress disorder. Three interventions offered education and/or communication with specialist nurses for cardiac patients. Three interventions were specifically for young people or children: one for pain self management by children in hospital, one for those with cystic fibrosis, and one for the management of encopresis.

### Reasons for Internet Delivery of Interventions

The reasons cited for using the Internet to deliver health interventions included the unique advantages of the Internet technology, reducing cost and increasing convenience for users, reducing health service costs, reaching isolated or stigmatized groups, timeliness of access to the Internet, need for user or supplier control of the intervention, and research-related reasons (Textbox).

Not all the studies in the early research papers mentioned the reasons for use of the Internet. Therefore, in the following analysis, the papers referenced are the papers for which the reason for Internet use was mentioned.

**Summary of findings**

**Reasons for Internet delivery:**

- Reducing cost and increasing convenience for users
- Reduction of health service costs
- Reduction of isolation of users
- The need for timely information
- Reduction of stigma
- Increased user and supplier control of the intervention

**Possible drawbacks of Internet interventions:**

- Potential for reinforcing the problems the intervention was designed to help
- May overcome isolation of time, mobility, and geography, but may be no substitute for face-to-face contact

**Elements of future evaluations:**

- Incorporate the cost not just to the health service, but also to users and their social networks
- Be alert to unintended effects of Internet delivery of health interventions, and include a comparison with more traditional modes of delivery

### Unique Advantages of the Internet Technology

There were 13 interventions studied [6,11,16,20,24,32,35, 37,41,45,52,57,60,67,71-73] for which the reason for Internet use was connected with the nature of the technology: reaching many people with just one posting, easy storage of large amounts of information, ease of updating information, providing personalized feedback, and the possibilities of broadband and video transmission. Two of these 13 studies [57,16] expressly valued the Internet for its ability to reach a maximum number of people at minimum cost. All these studies also give other reasons for Internet use.

### Reducing Cost and Increasing Convenience for Users

Reducing cost and increasing convenience for the user was given as a reason for delivery over the Internet in 20 of the interventions studied [20-23,29,32,39,43-48,50,52,53,56-58, 60-63,65,66,75]. These studies targeted a range of health issues. Various aspects of increased convenience to the user were mentioned, including saving the user time, requiring less effort from the user, being more accessible, and not requiring the user to attend a particular facility. One US study [43], reporting an intervention for women with breast cancer, stated users’ lack of money for a second opinion as one of the reasons for Internet delivery. Two studies advocated use of the Internet as it may reduce the loss of users from their maintenance programs for obesity [46,47].

### Reducing Health Service Costs

By using Internet delivery, 14 of the interventions studied [11,12,14,15,24,28-30,39,41,42,46,48, 57,58,60,65,67,68,72-75] aimed to reduce costs to health services or address a lack of provision. Of these, two studies, one on linking parents with their low-birth-weight babies in intensive care [41], and the other on the management of encopresis [60], specified reduction in health service cost as a reason for Internet use. The cost of service provision was also given as a reason by a number of other studies, but with slightly different emphases. One study saw the Internet as a cost-effective way of delivering an intervention to encourage physical activity in a broad range of people in many places [58]. Five of the interventions studied gave a lack of health service resources as their reason, two citing a lack of practitioners in CBT [11,12,42] and the others a lack of support for caregivers of those with Alzheimer’s disease [24,39] and AIDS [28-30]. All the above studies discussed a general lack of these services. None of the studies gave a specific localized service failure as the reason for Internet delivery, but two mentioned service failure more generally. One study [60] gave the lack of physicians trained in the treatment of encopresis as the reason for Internet delivery, while another study [67] aimed to reduce barriers to nutrition education due to general practitioners’ lack of skills and time. The authors of one other study [48] argued for Internet delivery because patients with diabetes have been found to have poor control despite specialist care, and their control may become even worse after the devolution of diabetes care to primary care services.

In six of the interventions studied, researchers wanted to avoid the cost to the health service of providing the intervention face-to-face, including psychological interventions for the treatment of depression [42], eating disorders[71-75],obesity [46-66], lack of physical activity [57,58], and a range of conditions (headache, tinnitus, panic attacks, and insomnia) [11-13].The authors of the latter intervention studied also argued that Internet delivery increases access to an otherwise costly therapy [14,15]. Another study [19] explicitly addressed inequalities of health care, suggesting that Internet delivery helped to overcome inequalities of access to health services.

### Reaching Isolated Groups

Dimensions of isolation were given as reasons for using the Internet in 13 of the interventions studied [18,19,21,25-30, 33,34,37,43,49,52,53,75]; 5 stated geographical isolation as their reason. These studies included interventions for rural women with chronic disease [33] or diabetes [34], an intervention for women with breast cancer [43], a cognitive behavioral program for eating disorders [75], an intervention for people with post-traumatic stress disorder [53], and an intervention for children with cystic fibrosis [52]. The authors of the latter study also mentioned the advantage of providing peer support without the risk of cross infection that can be life threatening for children with cystic fibrosis.

In other studies, the cause of the isolation was not geographical. One [28-30] stated the isolation of people living with HIV/AIDS as a reason for Internet delivery. Several studies cited the isolation of caregivers who were unable to easily go out, such as those living with an Alzheimer’s sufferer [21,25-27] and young mothers with children at home [37]. The physical immobility of individuals, including fatigue and disability, was mentioned in interventions focused on people with HIV/AIDS [28] or breast cancer [43] and on children with cystic fibrosis [52].

Several studies mentioned that Internet delivery enabled users to be in contact with people with similar health issues and so receive support. The implication was that this would be unlikely to happen otherwise as the condition was rare or restricting, for example, children in pain [49], children with cystic fibrosis [52], young mothers [37], people living with AIDS [28-30,43], and people with type 2 diabetes [18,19].

### Reaching Stigmatized Groups

The researchers of 11 interventions saw the Internet as a way of reaching people suffering from conditions that caused them to feel embarrassed or stigmatized [18,19,22,24, 28,30,31,37,43,45,46,57,60,73-75]. The anonymity of Internet delivery was a reason for using the Internet in the following interventions: an intervention for mental health problems [31], in which the authors considered stigma to be a problem; an intervention for people living with type 2 diabetes [18,19], in which the authors suggested that anonymity prevented people from being judged on the basis of their appearance; two interventions to improve the self-care of people living with AIDS [28,30,45]; an intervention for young women at risk of eating disorders [73-75]; and a support intervention for young mothers [37]. Three studies suggested that Internet delivery avoids embarrassment about the health issue for which the intervention was used. One of these was for breast cancer [43], the second referred to embarrassment about failure to lose weight in an obesity intervention [46], and the third was aimed at child encopresis [60]. One study of a support system for caregivers of patients with Alzheimer’s disease [22,24] and one study of an intervention to encourage physical activity [57] suggested that Internet delivery encourages openness of communication.

### The Timeliness of Access to the Internet

Several interventions [12,21,22,25,28-30,37,43] mentioned the need for timely information and advice as a reason for Internet delivery, including interventions to support Alzheimer’s caregivers [21,22,25], people with AIDS [28-30], young mothers [37], those with breast cancer [43], headache sufferers [12], and an intervention encouraging physical activity [57,58]. The suggestion was that people need information or advice at a time of crisis, for example, when their child is ill or when they are making a decision such as a change in treatment or their own behavior. The continuous access provided by the Internet was seen as helpful in these situations.

### User Control of the Intervention

Many authors advocated use of the Internet because users could take control of the intervention [11,21,32,37,45,48,58,60, 63,67,72,74], tailoring the information they received to their own needs. This included interventions for Alzheimer’s caregivers [21], those with HIV/AIDS [45], a glucose modeling tool for type 1 diabetes [48], and an intervention promoting physical activity [58]. Other studies advocated use of the Internet because users could use the intervention at their own pace. These included CBT for depression [32] or tinnitus [11], an educational program for cardiac patients [63], peer support interventions providing young mothers with support [37] or facilitating weight loss [67], an intervention for those at risk of eating disorders [72,74], and an intervention for encopresis [60].

### Supplier Control of the Intervention

For some interventions that delivered CBT as a self-help program, the Internet was seen as a potentially appropriate mode of delivery for such a structured, evidence-based intervention [12-15,32,42,57, 58,66,71-74]. One author stated that Internet delivery was superior to professional psychologists in delivering structured and standardized interventions [42]. However, in delivering these structured programs, the studies supplemented the standardized intervention through individualized email feedback, tailored information, online peer support, or a combination of the three.

### Research-Related Reasons

Almost all authors justified the evaluation of Internet-delivered interventions by saying that they need evaluating or adapting for specific populations. Six studies give this as their only reason [38,40,54,55,59,61]. Most studies give examples of successful Internet-delivered interventions to support their own research. However, one study gives, as its only reason for Internet-delivered intervention, that the intervention or a similar intervention had been useful in other studies [40]. One study [54] questions whether face-to-face and online support groups for those with breast cancer would work together, and gives this question as the only reason for delivering the intervention via the Internet.

A few studies did not give a research-related reason for evaluating an Internet-delivered intervention. These studies were descriptive accounts of an intervention [33,48,51] or were evaluating the use of an Internet-delivered intervention that was in response to a specific health service–related problem [60].

### Other Reasons

The following reasons, alongside others mentioned above, were also given for delivery of an intervention via the Internet:

- poor information received by patients from health professionals [48]
- novelty [57,58]
- attractiveness of the Internet to young people and children [51]
- online communication as one of the main forms of communication used by young people [60]

## Discussion

We have reviewed many pioneering studies evaluating Internet use for the delivery of health care interventions and found a variety of reasons for delivering interventions through the Internet. All the interventions have been, or could be, delivered by other means. For example, support groups for isolated individuals can use more established means of communication such as telephones and post, and therapeutic programs can be delivered face-to-face. The key differences between non-Internet delivered interventions and those delivered via the Internet relate to time and place. For example, Internet support groups enable quick communication between many isolated individuals, and Internet-delivered therapeutic interventions can be taken up at any time and anywhere with Internet access.

Our literature search strategy was designed to systematically identify the majority of eHealth intervention studies meeting our inclusion criteria. However, as a qualitative analysis that aimed to explore the motivations for delivering such interventions online, it was not necessary to undertake an exhaustive search for every single eHealth study ever published in any language. This contrasts with the methodology of quantitative meta-analysis, which requires the identification of all possible studies to produce one summary result. We believe that our qualitative thematic approach met our objective and was both rigorous and repeatable. Qualitative methods of research synthesis are a relatively new area and can be very valuable in identifying lessons for future work, particularly as they do not focus solely on the results on previous studies, but also consider other factors such as the researchers’ motivations. Our criteria for inclusion and exclusion of studies were designed to maintain the focus of the review on the added value from use of the Internet. Hence, they took account of the key characteristics of the Internet, particularly its networking potential and accessibility. Thus, our criteria differed from definitions of *eHealth*, for example, by excluding telemedicine [76] and general public access [77].

At this early stage of development, researchers should give careful thought to the reasons for using the Internet for any particular intervention. We should try to understand the unique advantages and disadvantages of Internet delivery of health care and in what circumstances Internet use could contribute most effectively to improving health. For example, why might speedier communication and flexibility of location enhance the effectiveness of the intervention? Answers may include, for example, overcoming inequalities of access to health services or encouraging openness of communication. However, to clarify the added contribution of Internet delivery over more traditional forms of delivery, evaluations should include a direct comparison between Internet-delivered interventions and those delivered by the most effective of available conventional means. Such evaluations will enable us to understand the effect of the real differences between the interventions. Few studies in our review undertook such a direct comparison.

Failing to undertake such a direct comparison may result in the failure to identify and quantify situations where face-to-face delivery is better than Internet delivery. For example, among the many studies of structured behavioral programs using Internet delivery, only one intervention [46,47] compared the benefits of this delivery method with time-intensive face-to-face therapy, and another compared it with a classroom-based intervention [70,72]. A systematic review comparing the effectiveness of Web-based and non-Web–based interventions [9] included, apart from the above two interventions, no other trials in which Web-based interventions had been compared to intensive face-to-face interventions. Undertaking an evaluation of Internet-delivered intervention without comparison may inappropriately encourage a reduction of the availability of the effective face-to-face intervention. This would work against the original motivation of the research to increase access to an effective intervention.

The design, delivery, and evaluation of an Internet-delivered intervention also need to consider the following questions: What may be the unintended harmful consequences of Internet delivery? What may be the negative effects of speedier communication and flexibility of location? For example, it is possible that providing low-cost Internet-based support for groups that are not currently provided with adequate support, such as caregivers of those with Alzheimer’s disease, may reinforce the low priority of these groups for health and social services and thus increase their isolation. Providing an intervention via the Internet for individuals living with a health problem they feel is stigmatized could have the unintended consequence of the issue being less talked about outside the anonymity of the Internet and thus reinforcing the stigma (see Textbox). Although identifying such unintended consequences was not an aim of this study, it was notable that we did not identify any reports of such consequences in the papers reviewed.

Evaluations of Internet-delivered interventions should aim to ensure that they include both the benefits and potential harms of the mode of delivery for all those affected by it. For example, an economic evaluation should include not only the cost of the Internet intervention, but also costs to health services, specific services, users, and their social networks. The studies reviewed rarely included an evaluation of such indirect costs.

Although the Internet can overcome isolation of time, mobility, and geography, it may be a poor substitute for face-to-face contact with real people. The balance between use of the Internet and face-to-face contact should be carefully considered in each circumstance. This applies to structured interventions such as CBT as well as to more flexible interventions such as peer-to-peer support. In designing an evaluation, researchers should be aware that Internet-based contact may be providing something different than face-to-face contact and should seek to assess these potentially different effects (see Textbox).

A number of studies gave no reason for use of the Internet as the mode of delivery beyond stating that it exists and needs evaluating. Now that the field of Internet- delivered interventions is established, future researchers should carefully consider how the networking provided by Internet delivery may enhance the effect of an intervention. This should involve exploratory work and more explicit use of existing theory and modeling [78].

The pioneering researchers who undertook the studies reviewed in this paper were often looking to the Internet for a way to help resolve some of the current difficulties and dilemmas of health care. These included the provision of equal access to health care, limitations on resources for health care, changing roles of health professionals, and changing needs for particular skills. Exploring the possible benefits of using the Internet to address these issues is important, but it is also important to make a meaningful comparison between using the Internet and using other more traditional ways of addressing the issues. Future research will hopefully shed more light on the benefits and disadvantages of Internet use particular to this mode of delivery.

## Appendix 1

**Table 1:** Summary table of reviewed studies

**Author**	**Year**	**Ref**	**Country**	**Health Condition**	**Description of Intervention**	**Networked Features**	**Reasons Given by Authors for Using Internet**
Andersson	2004	[11]	Sweden	Tinnitus	**CBT for headache, tinnitus, panic disorder, insomnia:** 6-module online self-help program based on cognitive behavioral therapy with email support from a trained therapist	Participants complete online progress reports; therapist responds by email	Advantage of technology Cost for health services User control of intervention
				Headache			
Andersson	2003	[12]					
				Tinnitus			
							Cost for health services Supplier control of intervention Timely information/advice
				Panic disorder			
Andersson	2002	[13]					
				Panic disorder			
Carlbring	2001	[14]					
							Supplier control of intervention
				Headache			
Carlbring	2003	[15]					
				Insomnia			
							Cost for health services Supplier control of intervention
Strom	2000	[16]					
							Cost for health services Supplier control of intervention
Strom	2004	[17]					
							Advantage of technology
							Research related only
Barrera	2002	[18]	United States	Type 2 diabetes	**D-net:** Internet-based self management program for type 2 diabetes with online feedback, professionally moderated but peer-directed message board, and access to professional coach	Message boards, chat facility (peer-to-peer and peer-to-professional)	Reaching isolated groups Reaching stigmatized groups
Glasgow	2003	[19]					
							Cost for health services Reaching isolated groups Reaching stigmatized groups
McKay	2002	[20]					
							Advantage of technology Cost for users
Bass	1998	[21]	United States	Alzheimer’s disease	**ComputerLink for Alzheimer’s caregivers:** information, communication, and resource center with nurse-led online support group (message board) with email facility, decision support system, encyclopedia, and links to quality websites	Message boards, email facility (peer-to-peer and peer-to-professional)	Cost for users Reaching isolated groups Timely information/advice User control of intervention
Brennan	1991	[22]					
							Cost for users Reaching stigmatized groups Timely information/advice
Brennan	1992	[23]					
Brennan	1994	[24]					
							Cost for users
Brennan	1995	[25]					
							Advantage of technology Cost for health services Reaching stigmatized groups
Casper	1995	[26]					
McClendon	1998	[27]					
							Reaching isolated groups Timely information/advice
							Reaching isolated groups
							Reaching isolated groups
Brennan	1991	[28]	United States	HIV/AIDS	**ComputerLink for people living with AIDS:** information, communication, and resource center with nurse-led online support group (message board) with email facility, decision support system, encyclopedia, and links to quality websites	Message boards, email facility (peer-to-peer and peer-to-professional)	Cost for health services Reaching isolated groups Reaching stigmatized groups Timely information/advice
Brennan	1994	[29]					
Flatley-Brennan	1998	[30]					
							Cost for users Cost for health services Reaching isolated groups Timely information/advice
							Cost for health services Reaching isolated groups Reaching stigmatized groups Timely information/advice
Chang	2001	[31]	United States	Mental health	**Mental health support for Asian-American men:** online support group moderated by Asian-American counselor	Message boards	Reaching stigmatized groups
Christensen	2002	[32]	Australia	Depression	**MoodGym:** online self-help program based on cognitive behavioral therapy	Participants complete online feedback sheets	Advantage of technology Cost for users User control of intervention Supplier control of intervention
Christensen	2004	[6]					
							Advantage of technology
Cudney	2000	[33]	United States	Chronic illness	**Women to Women:** nurse-led online support group for rural women with chronic illness; 1 subgroup with diabetes only	Message boards, email and chat facility to other peers and nurse	Reaching isolated groups
							Reaching isolated groups
Smith	2001	[34]					
Delgado	2003	[35]	Canada	Heart disease	Heart failure Internet communication tool	Email between patients and health professionals	Advantage of technology
							Research related only
Wu	2005	[36]					
Dunham	1998	[37]	Canada	Young mothers	**Support for young mothers:** peer-led online support group	Message boards, email facility, and teleconferencing	Advantage of technology Reaching isolated groups Reaching stigmatized groups Timely information/ advice User control of intervention
Feil	2003	[38]	United States	Smoking cessation	**Smoking cessation:** Web-based structured intervention and support program hosted by a para-professional ex-smoker	Message boards, email and ask-an-expert facility	Research related only
Glueckauf	2003	[39]	United States	Alzheimer’s disease	**Support for Alzheimer’s caregivers:** Web- and phone-based caregiver education and support program	Video-linked classes, peer-to-peer chat, and message boards	Cost for users Cost for health services Reaching isolated groups
Gomez	2002	[40]	United Kingdom/ Spain	HIV/AIDS	**Self-monitoring tool for people with AIDS:** Web-based recording and feedback system to enable self-care at home	Email ask-an-expert function based on patient-entered data	Research related only
Gray	2000	[41]	United States	Low-body-weight infants	**BabyCareLink:** education and communication tool for parents of children in intensive care	Reports/images of child, parent- ICU staff communication	Advantage of technology Cost for health services
Greist	2000	[42]	United States/ United Kingdom	Depression	**COPE:** Web-based (computer-enabled interactive voice re­sponse system) cognitive behavioral therapy for depression	Sends records and emergency signals to clinician	Cost for health services Supplier control of intervention
Gustafson	1993	[43]	United States	Breast cancer	**CHESS:** integrated information, referral, decision, and social support program for women with breast cancer	Facilitated online support group, ask-the-expert function	Cost for users Reaching isolated groups Reaching stigmatized groups Timely information/advice
Gustafson	2001	[44]					
							Cost for users
Gustafson	1999	[45]	United States	HIV/AIDS	**CHESS:** integrated information, referral, decision, and social support program for people with AIDS	Facilitated online support group, ask-the-expert function	Advantage of technology Cost for users Reaching isolated groups Reaching stigmatized groups User control of intervention
Harvey-Berino	2002	[46]	United States	Weight loss	**Weight loss program:** Web-based weight maintenance program following classroom-based weight loss intervention	Meetings with video-linked educator, chat room, message board, email facility	Cost for users Cost for health services Reaching stigmatized groups
Harvey-Berino	2002	[47]					
							Cost for users
Hejlesen	2000	[48]	Denmark	Type 1 diabetes	**DIASNet:** Web version of online modeling device used for self-management, communication, and education	Can be jointly used by patients and health professionals	Cost for users Cost for health services User control of intervention Poor info from professionals
Holden	2002	[49]	United States	Pain in children	**StarbrightWorld:** commercially developed interactive computer network for hospitalized children	Peer-to-peer emails, video links, chat rooms, bulletin boards	Reaching isolated groups
Hudson	1999	[50]	United States	Young mothers	**Social support for young mothers:** nurse-led email network providing health information and support	Email network (peer-to-peer and peer-to-nurse)	Cost for users Reaching isolated groups
Iafusco	2000	[51]	Italy	Type 1 diabetes	**Support group for teenagers with type 1 diabetes:** chat room with weekly meetings moderated by diabetologist	Chat room	Attractive to young people
Johnson	2001	[52]	United States	Cystic fibrosis	**Teen Central:** online support group for teenagers with cystic fibrosis	Moderated message boards, free “graffiti wall,” email facility	Advantage of technology Cost for users Reaching isolated groups
Lange	2003	[53]	Nether-lands	Post-traumatic stress disorder	**Interapy:** Internet-based cognitive behavioral writing program for people suffering from post-traumatic stress	Communication with therapists who read submitted writings and tailor standardized feedback	Advantage of technology Cost for users Reaching isolated groups
Lieberman	2003	[54]	United States	Breast cancer	**Support group for women with breast cancer:** electronic support group led by experienced cancer support facilitator	Weekly sessions, newsgroup, 24-hour chat room facility	Research related only
Lorig	2002	[55]	United States	Back pain	**Support group for back pain:** email discussion group with 2 professional moderators and 3 content experts	Email listserv	Research related only
Mahoney	1998	[56]	United States	Alzheimer’s disease	**Reach for TLC:** computer-mediated voice mail system to provide support and education for caregivers	Voice mail bulletin board, ask-the-expert facility	Cost for users
Marshall	2003	[57]	United States/ Australia	Physical activity	**Physical activity program:** online, workplace-based interactive behavioral change program	Email based on motivational stage and personalized goals	Advantage of technology Cost for health services Reaching stigmatized groups Timely information/advice Supplier control of intervention Novelty
Napolitano	2003	[58]					
							Cost for users Cost for health services Timely information/advice User control of information Supplier control of information Novelty
Pierce	2002	[59]	United States	Stroke	**Caring-Web:** nurse-led Web-based support group for caregivers of stroke victims	Email contact to nurse, email listserv (peers and nurse)	Research related only
Ritterband	2003	[60]	United States	Encopresis	**U-Can-Poop-Too:** Web-based enhanced toilet training for children with encopresis and their parents	Personalized homepage, follow-up sessions based on modules completed	Cost for users Cost for health services Reaching stigmatized groups User control of intervention Attractive to children
Robinson	2001	[61]	United Kingdom	Bulimia	**E-mail therapy for bulimia:** email treatment conducted by 2 clinicians experienced in eating disorders	Participants emailed diaries to which therapists responded	Research related only
Ross	2004	[62]	Canada	Heart disease	**Web-based Online Medical Record:** access to records and communication tool for patients with congestive heart failure	Messaging system between patients and cardiac nurses	Cost for users
Southard	2003	[63]		Heart disease	**Web-based educational program:** nurse-led educational program for secondary prevention of heart disease	Messaging between patients and nurses/dietitians	Cost for users User control of intervention
Takahashi	1999	[64]	Japan	Smoking cessation	**Quit Smoking Marathon:** smoking cessation program delivered through daily guidance emails	Message forum for participants, doctors and ex-smokers	None – description only
Tate	2001	[65]	United States	Weight loss	**Weight loss program:** Web-based behavioral weight loss program with email follow-up for those at risk of diabetes	Message board; participants submit diaries and weight; counselors respond by email	Cost for users
							Advantage of technology Cost for users Cost for health services Supplier control of intervention
Tate	2003	[66]					
Verheijden	2004	[67]	Netherlands	Weight loss	**Weight loss program:** peer support intervention to reduce fat consumption in those at risk of heart disease	Bulletin board for peer-to-peer communication and social support	Advantages of technology Cost for health service User control of intervention
Winzelberg	2003	[68]	United States	Breast cancer	**Support group for women with breast cancer:** Web-based social support group moderated by mental health professional	Message board with weekly discussion topic	Cost for users Supplier control of intervention
Dev	1999	[69]	United States	Eating disorders	**Student Bodies:** CD-ROM behavioral program plus Web-based counselor-led support group for students at risk of eating disorders	Moderated weekly discussion group (message board or email)	Research related only
							Research related only
Celio	2000	[70]					
							Advantage of technology Cost for health services Supplier control of intervention
Winzelberg	1998	[71]					
Winzelberg	2000	[72]					
							Advantage of technology Cost for health services User control of intervention Supplier control of intervention
Zabinski	2001	[73]					
Zabinski	2001	[74]					
							Advantage of technology Cost for health services Reaching stigmatized groups Supplier control of intervention
Zabinski	2003	[75]					
							Cost for health services Reaching stigmatized groups User control of intervention Supplier control of intervention
							Advantage of technology Cost for users Cost for health services Reaching stigmatized groups Reaching isolated groups

## Abbreviations

AIDS: acquired immunodeficiency syndrome
CBT: cognitive behavior therapy
HIV: human immunodeficiency virus
